# GlideRite® rigid stylet vs Flex-it® articulated stylet for intubation with videolaryngoscopy in patients with semi-rigid cervical collar: a randomized trial

**DOI:** 10.1016/j.bjane.2026.844836

**Published:** 2026-09-24

**Authors:** John Viet Nguyen, Eric Zhu, Said Dababneh, Pierre Drolet, Marie-Ève Bélanger, Rami Issa, Valerie Zaphiratos, Nadia Godin, Jean S. Bussières, Mihai Georgescu, Issam Tanoubi

**Affiliations:** a Université de Montréal, CIUSSS de l’Est-de-l’Île-de-Montréal, Maisonneuve-Rosemont Hospital, Department of Anesthesiology and Pain Medicine, Montréal, QC, Canada; b Université de Montréal, Faculty of Medicine, Montréal, QC, Canada; c Université de Montréal, Medical Simulation Centre, Centre d’Apprentissage des Attitudes et Habiletés Cliniques (CAAHC), Montréal, QC, Canada; d Institut Universitaire de Cardiologie et de Pneumologie de Québec (IUCPQ), Department of Anesthesiology and Intensive Care, Québec City, QC, Canada; e Université de Montréal, Centre de Recherche en Pédagogie de la Santé (CRPS), Montréal, QC, Canada

**Keywords:** Airway management, Immobilization, Intubation, intratracheal, Laryngoscopes, Laryngoscopy, Randomized controlled trial

## Abstract

**Background:**

Compared with the GlideRite® rigid stylet used with the GlideScope® hyperangulated blade, the Flex-it® stylet permits dynamic orientation of the tracheal tube toward the glottis. This randomized trial compared GlideRite and Flex-it stylets regarding GlideScope intubation duration in patients wearing a semi-rigid cervical collar, simulating conditions of cervical instability.

**Methods:**

Eighty adult patients undergoing elective surgery were randomized to tracheal intubation using the GlideScope with either the GlideRite or the Flex-it stylet. A semi-rigid cervical collar was applied following anesthesia induction. The primary endpoint was intubation duration in seconds. For our modified Intention-To-Treat (mITT) analysis, failed intubations were assigned the maximum duration of 180 seconds. Secondary outcomes included tracheal tube handling duration, intubation failure, Intubation Difficulty Scale, and ease of stylet removal.

**Results:**

Seventy-five patients were included in the mITT analysis (n = 38 for Flex-it, 37 for GlideRite). Intubation duration was longer when using Flex-it compared with GlideRite (median [IQR]: 88 [65‒180] vs. 49 [39‒61] s) with a median difference of 37 s (95% CI: 22 to 64; p < 0.0001). Tube handling duration was longer with Flex-it (median [IQR]: 48 [33‒66] vs. 34 [25‒39] s; p = 0.007). Failure rate was significantly higher with Flex-it (15/38, 39.5%) than with GlideRite (2/37, 5.4%; p < 0.001). Intubation Difficulty Scale score was higher for Flex-it (median [IQR]: 6 [5‒7] vs. 2 [2‒5]; p < 0.001).

**Conclusion:**

In elective patients with simulated cervical instability, videolaryngoscopy with the Flex-it stylet resulted in longer intubation, greater difficulty, and higher failure rate compared with the GlideRite stylet.

## Introduction

The GlideScope® video laryngoscope (Verathon® Medical Inc., Bothell, WA, USA) has a hyperangulated blade that improves glottic view over direct laryngoscopy. However, this angulation can cause difficulties when positioning the Tracheal Tube (TT), requiring routine use of an intubation stylet. A stylet mimics the curvature of the GlideScope blade, directing the TT toward the glottic aperture.^1^ The GlideRite® (Verathon® Medical Inc., Bothell, WA, USA) is a reusable rigid metal stylet with a 90° curvature and a radius origin of 6 cm, designed for GlideScope intubation. Nonetheless, GlideRite was not superior to a malleable stylet in elective patients without signs of difficult intubation, when used by either experienced^2^ or novice^3^ anesthesiologists. The Flex-it® (Parker Medical, Highlands Ranch, CO, USA) is a disposable plastic articulated intubation stylet, enabling operators to vary the angulation (Fig. 1) to orient the TT tip between the vocal cords, facilitating advancement down the trachea. It is also more pliable than GlideRite, potentially reducing tissue trauma during laryngoscopy. The Flex-it and GlideRite stylets have rarely been compared during videolaryngoscopy with a hyperangulated blade, especially in non-manikin-based difficult airway situations.

**Figure 1:**
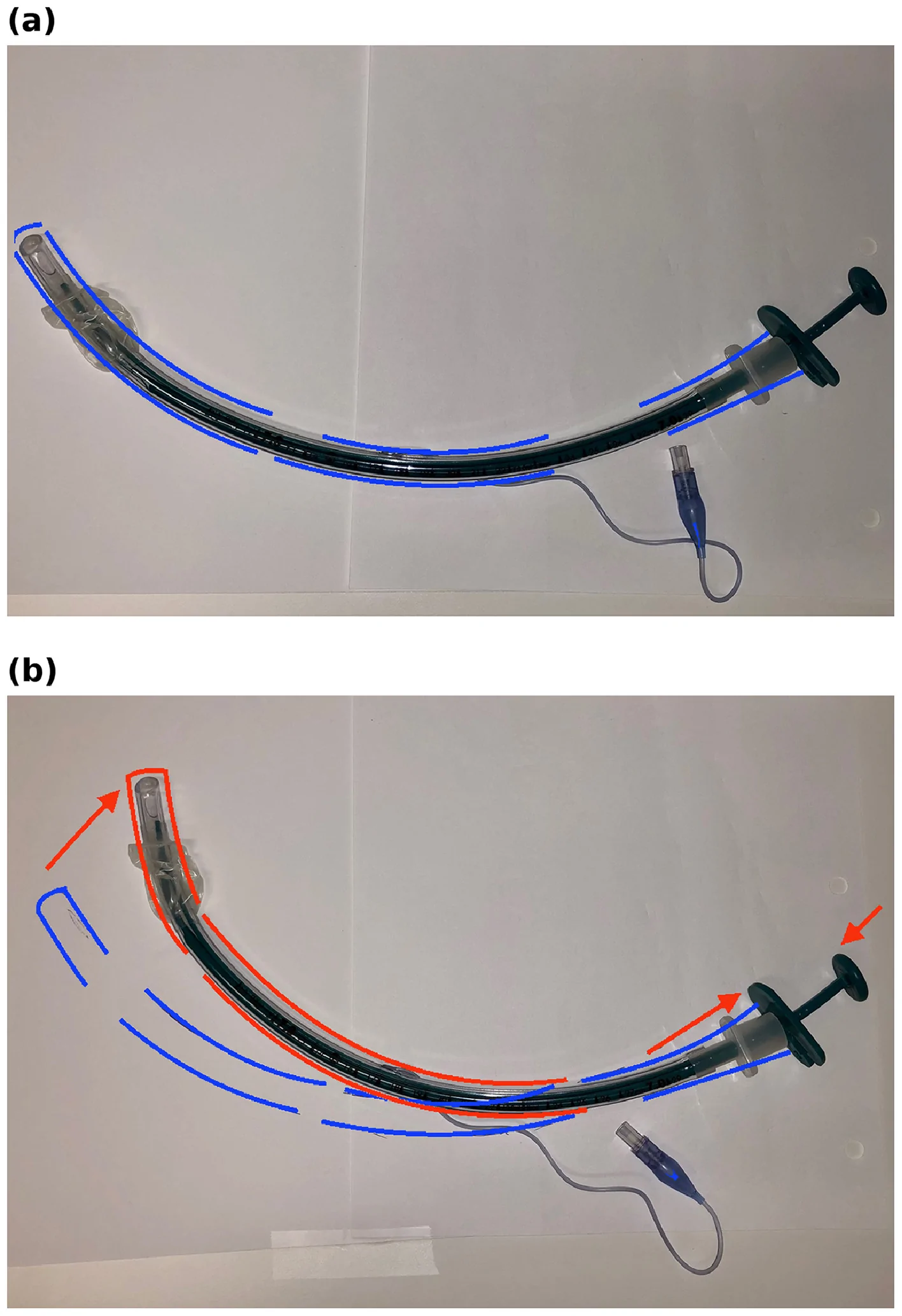
Impact of the Flex-it stylet tensioning on the curve of the tracheal tube. Flex-it released (a); Flex-it with tension (b).

In this randomized trial, we hypothesized that intubation duration would differ between the GlideRite and Flex-it stylets when used with the GlideScope hyperangulated blade in patients wearing a semi-rigid cervical collar. Secondary outcomes included TT handling duration, intubation failure rate, ease of intubation, hemodynamic response, and side effects.

## Methods

This was a single-center, parallel-group, randomized controlled trial conducted at Maisonneuve-Rosemont Hospital from 14 June 2014 to 17 November 2016. The study is reported in accordance with the Enhancing the QUAlity and Transparency Of health Research (EQUATOR) CONsolidated Standards Of Reporting Trials (CONSORT) guidelines.^4^ Our institutional research ethics board approved the study protocol for this trial on 12 May 2014 (IRB approval number: 14013). The trial was registered before patient enrollment on 29 May 2014 (ClinicalTrials.gov registration: NCT02177240). Written informed consent was obtained from all participants.

### Study population

Inclusion criteria were American Society of Anesthesiologists (ASA) physical status I or II, age 18 to 65 years, body mass index of ≤ 35 kg.m^−2^, and elective surgery under general anesthesia requiring a TT. Exclusion criteria were Mallampati score of ≥ 3, mouth opening < 2.5 cm, thyromental distance < 6 cm, limited cervical mobility, upper respiratory tract abnormalities (e.g., trauma, radiotherapy), difficult intubation history, rapid sequence induction, or contraindication to GlideScope or cervical collar.

### Procedure

Simple randomization with 1:1 allocation was performed using an electronic randomization list (randomizer.org) generated by an independent research assistant not involved in patient enrollment, with codes kept in opaque, sequentially numbered envelopes. Each envelope was opened only after the patient had undergone anesthesia induction, immediately prior to intubation. Given the nature of the intervention, stylet type was revealed once the TT was handed to the operator. Therefore, while participants and outcome assessors were blinded, operators were not blinded to the intervention.

For each patient, the respiratory therapist prepared two TTs with the studied stylets, concealing them under a towel. Upon patient arrival, the respiratory therapist established intravenous access and started an infusion of lactated Ringer’s solution. Standard monitoring included pulse oximetry, non-invasive blood pressure, 5-lead ECG, Train-Of-Four (TOF) monitoring of neuromuscular blockade, and Bispectral Index (BIS) (Medtronic, Kirkland, QC, Canada). The attending anesthesiologist decided on additional monitoring and administered premedication with 1 mg intravenous midazolam unless contraindicated. Patients were preoxygenated to an end-tidal oxygen concentration of 85% before induction. The anesthesiologist induced the patient using propofol 1 to 3 mg.kg^−1^, sufentanil 0.1 to 0.3 μg.kg^−1^, and rocuronium 0.4 to 0.6 mg.kg^−1^. The respiratory therapist performed mask ventilation until the response to ulnar neurostimulation disappeared, determined by a TOF count of 0/4 (no muscle response to any of the four stimuli). The research assistant then applied a Philadelphia semi-rigid cervical collar (Össur Americas, Paulsboro, NJ, USA) and secured the patient’s head to the operating table with tape.

One of three participating anesthesiologists inserted the GlideScope to achieve a satisfactory glottic view. All intubations were performed using the same blade model in both study arms. No standardized instructions were provided to operators regarding technique. The respiratory therapist then provided the TT (7 mm for female patients, 8 mm for male patients) with either GlideRite or Flex-it stylet according to allocation. Only then did the operator learn the allocated stylet. They confirmed TT positioning using a satisfactory End-Tidal CO_2_ (ETCO_2_) waveform, then ventilated the patient according to standard practice. No changes to the study protocol or outcomes occurred after trial initiation.

The anesthesiologists had eight to ten years of practice and were proficient in both direct laryngoscopy and videolaryngoscopy. They completed a minimum of 10 GlideScope intubations with each stylet before participating, either on patients or on manikins.^5^

Removing the TT or GlideScope blade from the patient’s mouth before a successful intubation was considered a failed attempt. Between attempts, the patient was ventilated at the anesthesiologist’s discretion. Intubation failure was defined as a procedure requiring more than three attempts or exceeding 180 seconds in duration. Failed intubations were assigned the maximum time (180 seconds) for our modified Intention-To-Treat (mITT) analysis. In such cases, the attending anesthesiologist managed the airway and restored standard care.

During the procedure, the principal investigator filmed the patient’s monitors with a video camera (Handycam Camcorder, Sony Corporation, Tokyo, Japan) while the assistant verbally signaled events. The assistant said “blade” to signal the insertion of the blade into the patient’s mouth and “tube” to signal the operator’s handling of the tube. Capnograph waveforms were visible on the recordings. We did not capture the actual intubation procedure. This method allowed blinded assessment of timing outcomes.

### Primary outcome

The primary outcome was the intubation duration in seconds, defined as the interval from placing the GlideScope blade into the patient’s mouth until detection of ETCO_2_ ≥ 30 mmHg.

### Secondary outcomes

Secondary outcomes included TT handling duration in seconds (interval from TT insertion to detection of ETCO_2_ ≥ 30 mmHg), intubation failure (more than three attempts or longer than 180 seconds), number of attempts (any removal of the TT or the GlideScope blade from the patient’s mouth), Intubation Difficulty Scale (IDS) score, difficulty in removing the stylet (assessed on a 5-point Likert scale), postoperative hoarseness and sore throat (assessed immediately after the surgery and after 24 hours on a 5-point Likert scale), and glottic trauma incidence (blood stains on the TT after extubation). The IDS has seven parameters, providing a progressive, quantitative assessment of the complexity involved in both direct and indirect laryngoscopy.^6^ Although the IDS was developed for direct laryngoscopy and does not include parameters unique to videolaryngoscopy, it remains a clinically useful scale for evaluating intubation difficulty.^7^^,^^8^ In this study, the IDS was applied using the standard scoring method. An IDS score ≥ 4 indicates a moderately difficult intubation. We also compared blood pressure, heart rate, and oxygen saturation measurements before and 1 minute after intubation to evaluate the response to the procedure.

### Statistical analysis

#### Primary outcome

We performed statistical analysis using Prism 7 for macOS (GraphPad Software, La Jolla, CA). We analyzed time to successful intubation under an mITT framework. Failed intubations were assigned the maximum allowed duration of 180 seconds. Given the non-normal distribution of intubation duration (assessed using the D’Agostino-Pearson omnibus test), we summarized the data as medians with interquartile ranges and compared them between groups using the Mann-Whitney test. We used the Hodges-Lehmann estimator to quantify the median difference between groups, with corresponding 95% Confidence Intervals.

In addition, we performed time-to-event analysis using Kaplan-Meier methods to describe the temporal dynamics of successful intubation. Because failed intubations were assigned a fixed duration, this analysis is meant to be descriptive and hypothesis-generating. Survival curves were compared using the Gehan-Breslow-Wilcoxon test, which places greater weight on early events and was selected given the clinical relevance of early intubation success. Hazard ratios with 95% confidence intervals were estimated using the Mantel-Haenszel method and log-rank test.

#### Secondary outcomes

Because the TT handling duration was non-normally distributed, we chose to use the D’Agostino-Pearson test. It was then compared using the Mann-Whitney test and presented as median [IQR]. We compared the incidence of intubation failure and glottic trauma using the Pearson Chi-square test and presented them as percentages. We reported the Relative Risk (RR) and Absolute Risk Difference (ARD) with 95% confidence intervals. We used a Mann-Whitney test to analyze data on the number of attempts, IDS scores, stylet removal difficulty, and hoarseness or sore throat intensity and presented this data as median [IQR]. The blood pressure, heart rate and oxygen saturation measurements were presented as mean ± SD. As these parameters were compared at a single time point (1 minute after intubation), we used an unpaired *t*-test after confirming normality of the data using the Shapiro-Wilk test. The significance level was 0.05 for each hypothesis test.

#### Sample size calculation

Based on a study comparing the Flex-it stylet with a malleable guide during videolaryngoscopy,^9^ median [IQR] intubation duration was 15 s [8‒28]. We estimated the corresponding mean (SD) value using the method described by Hozo et al.^10^ at 16 seconds (5.7), which we used to calculate the sample size needed to demonstrate a 33% difference (5 s) between interventions in intubation duration, assuming the same SD for both. We estimated sample size using Bernard Rosner’s method^11^ and concluded that we needed 36 patients per intervention, with α = 0.05 and β = 0.20. We aimed to recruit 80 patients to compensate for exclusions or losses.

## Results

Seventy-five participants were included in the mITT analysis, with 38 patients in the Flex-it group and 37 patients in the GlideRite group (Fig. 2). Two patients in the Flex-it group and three in the GlideRite group were excluded from analysis because monitoring data could not be extracted. Patient characteristics are presented in Table 1. Demographic parameters and categorical variables are compared using the Absolute Standardized Difference (ASD) (Table 1).

**Figure 2:**
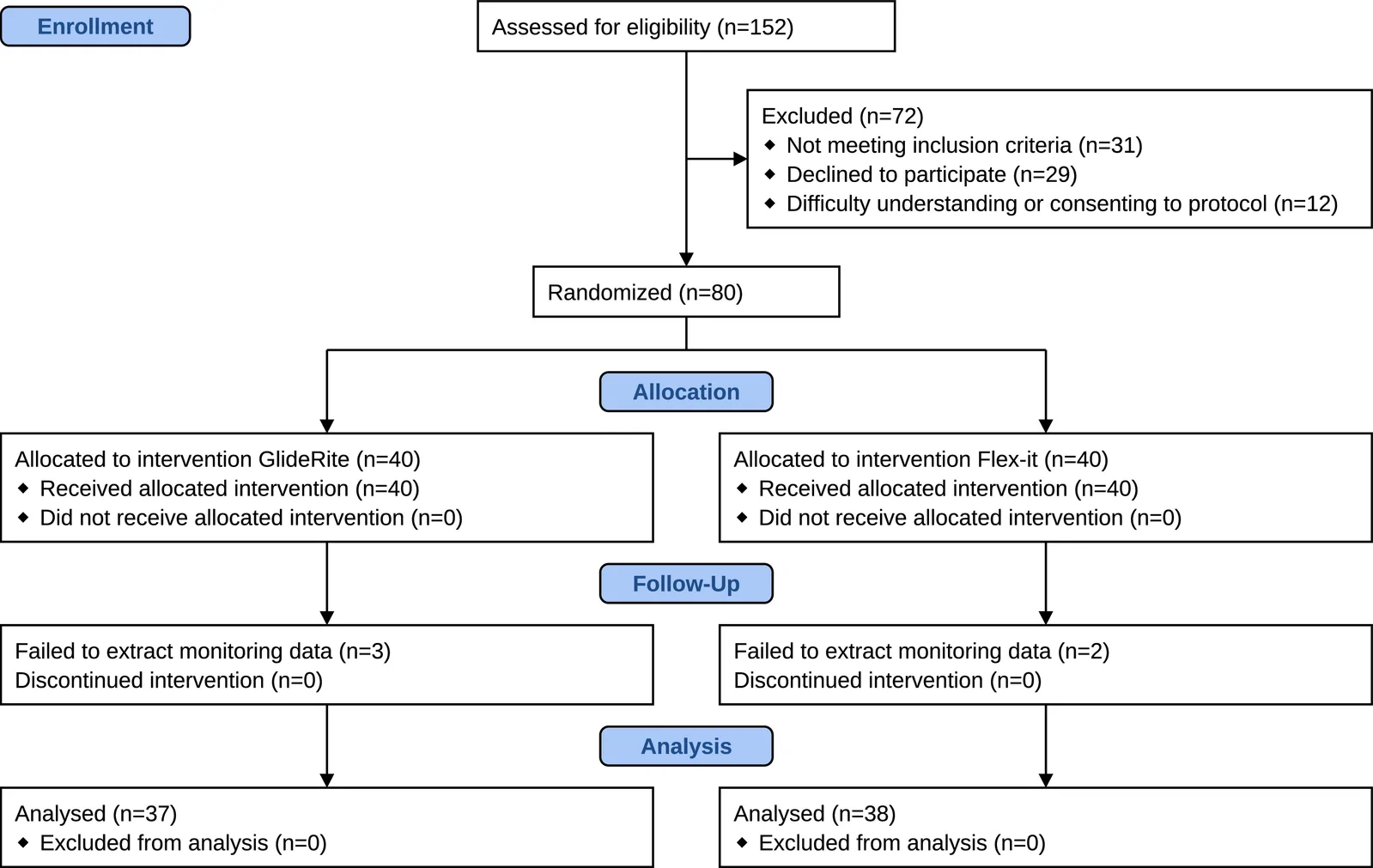
CONSORT flow diagram.

**Table 1:** Characteristics of patients intubated with GlideScope + GlideRite stylet (GlideRite) and with GlideScope + Flex-it stylet (Flex-it).

	GlideRite	Flex-it	Absolute standardized difference (ASD)
**Patients, n**	37	38	
**Age (mean ± SD)**	45 ± 12	50 ± 12	0.4
**Males (%)**	47	42	0.10
**Body Mass Index (kg.m^−2^, mean ± SD)**	25 ± 4.2	27 ± 4.7	0.44
**Edentulous patients (%)**	23	18	0.12
**American Society of Anesthesiologists (ASA) physical status (I/II)**	11/26	10/28	0.08
**BIS index before intubation (mean ± SD)**	32 ± 10	36 ± 13	0.38

### Primary outcome

Time distributions were markedly non-normal in both groups (D’Agostino-Pearson p < 0.0001). Median intubation duration was significantly shorter in the GlideRite group compared with the Flex-it group (median [IQR]: 49 [39‒61] vs. 88 [65‒180] s), with a median difference of 37 s (Hodges-Lehmann estimate, 95% CI: 22 to 64; p < 0.0001).

We examined time to successful intubation using Kaplan-Meier methods as an additional analysis (Fig. 3). Survival curves differed significantly using the Gehan-Breslow-Wilcoxon test (p = 0.0418), suggesting an early advantage with GlideRite. The hazard ratio did not differ significantly between groups (log-rank HR = 1.03, 95% CI: 0.65 to 1.62). This analysis was exploratory and should be interpreted as supportive rather than confirmatory of the primary analysis.

**Figure 3:**
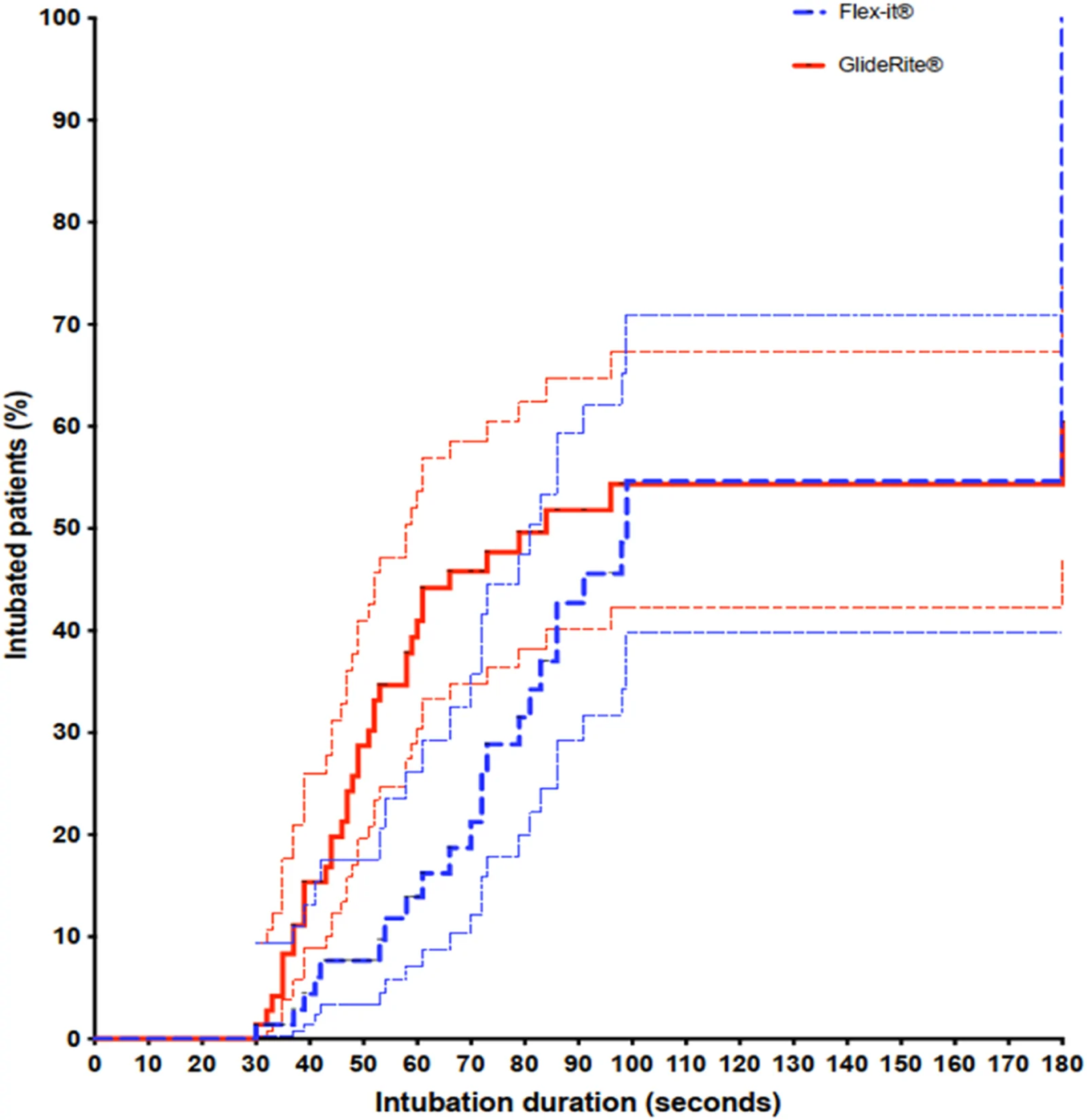
Kaplan-Meier curves show time to successful intubation for Flex-it (blue) and GlideRite (red). Failed intubations were assigned a duration of 180 seconds. The GlideRite group demonstrated faster intubation, particularly at earlier time points (Gehan-Breslow-Wilcoxon p = 0.0418).

### Secondary outcomes

TT handling duration was longer with Flex-it than with GlideRite (median [IQR]: 48 [33‒66] vs. 34 [25‒39] s) with median difference of 14 s (Hodges-Lehmann estimate, 95% CI: 3 to 25; p = 0.007). Failure rate was significantly higher with Flex-it (39.5%) than with GlideRite (5.4%) (RR = 7.30; 95% CI: 1.79 to 29.74, ARD = 34.07%; 95% CI: 16.90% to 51.23%, Number Needed to Harm [NNH] = 3; p < 0.001). Fewer attempts were required with GlideRite than with Flex-it (median [IQR]: 1 [1‒2] vs. 3 [1‒3]), Hodges-Lehmann median difference of 1, 95% CI: [0 to 2] (p < 0.001). IDS score was significantly lower with GlideRite (median [IQR]: 2 [2‒5] vs. 6 [5‒7]) with median difference of 3 (Hodges-Lehmann estimate, 95% CI: 1 to 4; p < 0.001). No serious adverse events occurred. Ease of stylet removal, hoarseness, sore throat, incidence of glottic trauma, blood pressure, and heart rate were not different between interventions. Oxygen saturation 1 minute after intubation was slightly higher with GlideRite (98.8 ± 0.9% vs. 98.3 ± 0.9%; p = 0.027) (Table 2).

**Table 2:** Primary and secondary outcomes for GlideRite and Flex-it interventions.

		GlideRite	Flex-it	p-value	Treatment effect: Difference between medians [95% CI] or difference between means ± SEM	Relative Risk (RR) [95% CI]	Absolute Risk Difference [95% CI]
**Primary**	Intubation duration (s, median [IQR])	49 [39‒61]	88 [65‒180]	< 0.0001	37 [22 to 64]		
**Secondary**	Tracheal tube handling duration (s, median [IQR])	34 [25‒39]	48 [33‒66]	0.007	14 [3 to 25]		
	Failed intubation (n, incidence %)	2, 5.4%	15, 39.5%	< 0.001		7.30 [1.79 to 29.74]	34.07% [16.90 to 51.23]
	Number of attempts for intubated patients (median [IQR])	1 [1‒2]	3 [1‒3]	< 0.001	1 [0 to 2]		
	Intubation Difficulty Scale score (median [IQR])	2 [2‒5]	6 [5‒7]	< 0.001	3 [1 to 4]		
	Stylet removal difficulty (median [IQR])	2 [1‒3]	2 [1‒3]	0.527	0 [0 to 1]		
	Hoarseness intensity (median [IQR])	1 [0‒1.25]	0 [0‒1]	0.465	0 [0 to 1]		
	Sore throat intensity (median [IQR])	0 [0‒1]	0.5 [0‒1]	0.730	0 [0 to 0]		
	Incidence of glottic trauma (n, incidence %)	1, 2.7%	3, 7.9%	0.317		2.92 [0.32 to 26.83]	5.19% [-4.85 to 15.23]
	Systolic blood pressure 1 min after intubation (mmHg, mean ± SD)	125 ± 30	141 ± 37	0.086	16.4 ± 9.4		
	Heart rate 1 min after intubation (bpm, mean ± SD)	85 ± 10	89 ± 12	0.164	4.4 ± 3.1		
	Oxygen saturation 1 min after intubation (%, mean ± SD)	98.8 ± 0.9	98.3 ± 0.9	0.027	0.59 ± 0.26		

Primary and secondary outcomes for patients randomized to GlideScope intubation with the GlideRite rigid stylet or the Flex-it articulating stylet. Data are presented as median [interquartile range] or mean ± standard deviation unless otherwise indicated. Treatment effects for continuous outcomes are expressed as the Hodges-Lehmann median difference [95% Confidence Interval] or as the difference between means ± Standard Error of the Mean (SEM). Treatment effects are expressed as absolute differences between groups. Binary outcomes are reported as number of events and incidence (%), with Relative Risk (RR) and Absolute Risk Difference (ARD) with 95% confidence intervals. Intubation failure was defined as a procedure exceeding 180 seconds or requiring more than three attempts; a failed attempt was defined as any removal of the tracheal tube from the patient's mouth or repositioning of the GlideScope blade. The Intubation Difficulty Scale score ≥ 4 indicates at least moderate intubation difficulty. Stylet removal difficulty was rated by the performing anesthesiologist on a 5-point scale (1 = very easy, 5 = very difficult). Hoarseness and sore throat were assessed by a blinded recovery room nurse on a 5-point Likert scale.

## Discussion

In this randomized trial, tracheal intubation with a hyperangulated GlideScope blade was significantly inferior when the Flex-it stylet was used compared with the GlideRite rigid stylet across the primary and principal secondary outcomes. Intubation duration was significantly longer with the Flex-it stylet. However, the most clinically meaningful finding was intubation failure, with the Flex-it group experiencing a failure rate of 39.5% versus 5.4% in the GlideRite group. The NNH was 3, indicating one additional failure for approximately every three patients intubated with the Flex-it. However, the wide confidence interval (RR: 95% CI 1.79 to 29.74) reflects the limited number of failures and warrants cautious interpretation of the precise magnitude of this effect.

Turkstra et al.^9^ compared the Flex-it to a malleable stylet and found that intubation was longer and more difficult with the Flex-it, consistent with our results. However, they did not report a comparable failure rate disparity.

Our findings contrast with those of Bak et al.^12^, who reported no difference in intubation duration between the GlideRite and Flex-it stylets in a simulated difficult airway, despite a subjective higher difficulty rating for the Flex-it stylet. Similarly, Sheta et al.^13^ found comparable intubation durations and success rates for both stylets in patients with normal airways using a GlideScope hyperangulated blade. Methodological differences may explain the divergence between these results and our own. Neither study reported intubation failure as a primary or secondary outcome. Thus, the high failure rate we observed using the Flex-it stylet may have gone undetected in smaller samples or less challenging airways. Meanwhile, Reus et al.^14^ reported a higher first-attempt success rate with the Flex-it when used with the McGrath® video laryngoscope. This was done across multiple simulated airway scenarios in manikins and may not be generalizable to our findings. Their findings may also reflect a device-specific interaction, as the McGrath employs a different blade geometry and may be more forgiving of variable stylet characteristics than the GlideScope blade.

Our results may be explained by mechanical differences between the stylets. The articulated design of the Flex-it was intended to provide dynamic modulation of the TT tip,^15^ theoretically offering an advantage over rigid stylets for indirect visualization of the glottis.^16^^,^^17^ Instead, we observed that the tension required to deflect the TT tip anteriorly caused irreversible deformation of the stylet. This caused it to effectively function as an unpredictable malleable stylet rather than a dynamic articulated device. Because the GlideScope hyperangulated blade requires reliable upward redirection of the TT tip to navigate the acute angle between the blade and the glottis, the excessive malleability of the Flex-it and its inability to maintain or restore its original curvature may explain both the prolonged intubation duration and high failure rate observed in our study. Consistent with this interpretation, studies performed with normal airways^13^ or blades with reduced angulation^18^ have not demonstrated the same inferiority, suggesting that the choice of stylet becomes clinically consequential under specific conditions that require reliable TT tip redirection. Our findings might vary if a less angulated blade was used, which requires fewer adjustments at the distal end.^18^

The combination of both prolonged intubation duration and higher failure rates carries meaningful clinical implications in situations where speed and reliability are paramount, such as in patients with suspected cervical instability. Based on our primary mITT analysis, the GlideRite stylet should be considered the preferred adjunct with the GlideScope hyperangulated blade in this patient population.

This study was conducted between 2014 and 2016. The protocol, endpoints, and analysis plan remain unchanged since trial registration. We acknowledge that videolaryngoscopy practice, technology, and familiarity have substantially evolved in the past decade. Hyperangulated videolaryngoscopy, including the GlideScope, has become widely adopted as a primary intubation technique.^19^ Contemporary anesthesiologists may have greater baseline familiarity with hyperangulated videolaryngoscopy than the participants in our trial. This could attenuate the magnitude of the effect observed, though it is equally plausible that the intrinsic mechanical limitations of the Flex-it stylet would persist.

### Limitations

This study has several limitations. First, since rigid stylets are more commonly used than malleable ones like Flex-it, operator familiarity with the GlideRite may be greater. A study comparing intubation duration with two different video laryngoscopes suggests that time to intubation by an operator converges by the sixth procedure.^5^ Although we standardized training to a minimum of 10 intubations with each stylet, performance bias favoring the GlideRite cannot be completely excluded. In particular, the markedly higher failure rate observed with the Flex-it stylet may partly reflect differential operator familiarity rather than an intrinsic limitation of the device itself. In addition, because no instructions were provided concerning technique, operator variability represents an additional consideration. The contribution of operator technique to the observed results cannot be determined.

Second, this trial excluded patients with predictors of anatomically difficult airway, including Mallampati score ≥ 3, mouth opening < 2.5 cm, thyromental distance < 6 cm, prior history of difficult intubation, and limited cervical mobility. Difficulty was therefore imposed extrinsically via cervical collar and head fixation, which does not replicate actual cervical instability encountered in clinical practice, nor does it reflect intrinsic anatomical challenges. This may underestimate the difference between stylets in true difficult airway scenarios involving distorted anatomy, trauma, hemorrhage, or other emergency scenarios. Consequently, our study design may have limited the ability to detect a potential advantage of articulated stylets, which theoretically permit greater adaptability in patients with anatomically unusual airways.

Third, all intubations were performed by experienced anesthesiologists. While this supports internal consistency, it limits generalizability to less experienced operators, in whom performance differences may be even more pronounced.

Fourth, the initial sample size calculation was based on parametric assumptions, whereas the primary analysis was ultimately conducted using non-parametric methods. As such, the estimated sample size should be interpreted as approximate. Moreover, the trial was not explicitly designed to detect the observed difference in failure rate. Intubation duration was chosen as the primary outcome because it is a commonly used primary endpoint for stylet comparison studies. Additional studies with intubation failure as a primary endpoint may be warranted.

## Conclusion

In elective adult patients without predictors of anatomically difficult airway, using a cervical collar to simulate conditions of cervical instability, intubation with the GlideScope hyperangulated blade using the Flex-it stylet was significantly slower and more difficult, and had a markedly higher failure rate than with the GlideRite stylet.

## Data availability statement

The de-identified dataset supporting the findings of this study has been deposited in Zenodo and is publicly available at 10.5281/zenodo.22004806.

## Declaration of generative AI and AI-assisted technologies in the manuscript preparation process

No artificial intelligence tools were used during the preparation of this work. The authors take full responsibility for the content of the published article.

## Authors’ contributions

John Viet Nguyen: Conceptualization; data curation; formal analysis; funding acquisition; investigation; methodology; validation; writing-original draft.

Eric Zhu: Formal analysis; validation; writing-review and editing.

Said Dababneh: Writing-review and editing.

Pierre Drolet: Writing-review and editing.

Marie-Ève Bélanger: Data curation; validation; visualization; writing-review and editing.

Rami Issa: Data curation; validation; visualization.

Valerie Zaphiratos: Validation; visualization; writing-review and editing.

Nadia Godin: Investigation; project administration; resources; supervision; validation.

Jean S. Bussières: Conceptualization; methodology; writing-original draft.

Mihai Georgescu: Writing-review and editing.

Issam Tanoubi: Conceptualization; data curation; formal analysis; funding acquisition; investigation; methodology; project administration; resources; supervision; validation; writing-original draft; writing-review and editing.

## Funding

This research did not receive any specific grant from funding agencies in the public, commercial or not-for-profit sectors.

## Conflicts of interest

The authors declare no conflicts of interest.
